# Chronic Reproductive Toxicity of Fomtec Enviro USP, a Fluorine-Free Firefighting Foam, to Northern Bobwhite (*Colinus virginianus*)

**DOI:** 10.3390/toxics13060474

**Published:** 2025-06-03

**Authors:** Anna S. Longwell, Farzana Hossain, Seenivasan Subbiah, Adcharee Karnjanapiboonwong, Jamie G. Suski, Todd A. Anderson

**Affiliations:** 1 Department of Environmental Toxicology, Texas Tech University, Lubbock, TX 79409, USA; anna.s.longwell@gmail.com (A.S.L.); fhjhumi@gmail.com (F.H.); seenivasan.subbiah@ttu.edu (S.S.); adcharee.karnjanapiboonwong@ttu.edu (A.K.); 2 EA Engineering, Science, and Technology, Inc., Hunt Valley, MD 21031, USA

**Keywords:** avian, fluorine-free AFFF, chronic toxicity values

## Abstract

Long-chain per- and polyfluoroalkyl substances (PFASs) have been the standard active chemicals in aqueous film-forming foams (AFFFs or firefighting foams) since the mid-1960s. Some characteristics of PFASs are environmental persistence and bioaccumulation. Non-fluorinated firefighting foams are an alternative to potentially reducing the ecological/environmental impact of PFAS-based AFFF. We used northern bobwhite (NOBO, *Colinus virginianus*) to test the ecotoxicity of one candidate (non-fluorinated) foam. Fomtec Enviro USP is a fluorine-free commercial AFFF used primarily for extinguishing Class B hydrocarbon fuel fires. Following a photostimulation phase to initiate egg laying, breeding pairs were exposed for 60+ days to 0.01%, 0.1%, and 0.25% Fomtec in drinking water. The endpoints of the study included survival, growth, and reproductive output. Water consumption was evaluated and used to determine the average daily intake (ADI) based on Fomtec components: sodium dodecyl sulfate or SDS (0.05, 0.15, and 0.32 mg/kg/day for the 0.01%, 0.1%, and 0.25% Fomtec exposures, respectively) and diethylene glycol monobutyl ether or DGMBE (0.49, 6.54, and 18.37 mg/kg/day for the 0.01%, 0.1%, and 0.25% Fomtec exposures, respectively). Over the 60 days, control females laid an average of 59 ± 0.8 eggs compared to 28 ± 9 (0.01% Fomtec exposure), 51 ± 4 (0.1% Fomtec exposure), and 56 ± 2 (0.25% Fomtec exposure); the number of eggs produced per hen was affected by exposure to the lowest Fomtec concentration. Hatching success was not significantly different among treatment groups, and it was within normal reproduction parameters for quail. Our findings in this avian model help to fill data gaps for non-fluorinated foam products, many of which have little toxicological information.

## 1. Introduction

PFASs are a class of over 4000 synthetic, highly fluorinated hydrocarbons that come in different chain lengths [1]. Since the 1950s, PFASs have gained vast application in consumer and industrial products. Some of the many uses of PFASs include stain-resistant coatings for textiles, upholstery, and carpeting and use in oil-resistant coatings, floor polish, and firefighting foams [2,3]. PFASs are stable, making breakdown through hydrolysis, photolysis, or microbial degradation of PFASs very difficult; many exhibit half-lives between 41 and 92 years [1,2]. Their many uses and persistence have led to the distribution of PFASs on a global scale [3]. With prolonged exposure, PFAS mixtures can disrupt endocrine functions and act as carcinogens [4,5]. Within tissues, the highest concentrations of PFASs are in the blood, liver, bile, kidney, and fat [1,6], and there is potential for maternal transfer of PFASs into eggs or other highly lipid-rich tissues [7].

Aqueous film-forming foams, known as AFFFs, are used to extinguish and prevent the re-ignition of hydrocarbon fires in public, commercial, and military firefighting operations [8]. Frequently, AFFFs are used as 3 or 6 percent aqueous solutions by volume [9]. Since the 1960s, the PFAS components of AFFFs have been detected in significant quantities, specifically surrounding airports and other large areas around military bases and firefighting training facilities [9]. As a result, soil and groundwater have become contaminated with high levels of PFASs and serve as a potential exposure source for wildlife populations. PFAS-containing AFFFs come in long and short chains; longer-chain PFASs are more bio-accumulative and toxic to many species [7]. In the 2000s, 3M started a global phase-out of long-chain PFAS (C6, C8, and C10) products for shorter-length (C4) chains as a start to finding alternatives to traditional AFFFs [10,11].

Fluorine-free AFFFs are a potential alternative to traditional AFFFs that contain PFASs. Fomtec Enviro USP is a commercial non-fluorinated foam used in Class A and B fires. It contains diethylene glycol monobutyl ether (DGMBE), C12-C14 sulfuric acid esters, C12-C14 alcohols ethoxylated sulfates, and C12-C14 amines N-oxides, and it is known to be 60% biodegradable in 28 days. While there are likely fewer environmental and ecological concerns with these new fluorine-free foams in terms of persistence and bioaccumulation, it is prudent to evaluate the ecotoxicity of these fluorine-free foam products given the absence of data. Acute toxicity may be the most relevant endpoint for these fluorine-free firefighting foams, as they contain chemical components that generally lack persistence and thus may be less likely to present a long-term hazard. Fomtec Enviro USP has an acute LD50 > 1561 mg/kg in adult NOBO [12].

Our objective was to obtain toxicology data regarding a formulated fluorine-free firefighting foam. We examined relevant endpoints of mortality (adult and chick survival), growth, and reproduction (egg production and hatching success). In addition, we used the results of a chronic study to determine chronic toxicity values based on the formulated product as well as the primary components of the foam, including tissue-specific values obtained with the aid of residue analyses. The majority of the research reported herein is based on the M.S. thesis research of Anna Longwell [13].

## 2. Materials and Methods

### 2.1. Study Design and Quail Husbandry

A toxicity test was performed using avian reproductive toxicology test protocols [14]. A total of 35 NOBO were purchased as the test species from TNT Game Birds (Lubbock, TX, USA). Birds were randomly assigned to treatments, separated into 15 male:female pairs, and housed in 15-compartment battery breeding pens (GQF Manufacturing, Savannah, GA, USA). The study design allowed for a weeklong acclimation period where deionized water and Purina^®^ Game Bird Breeder Layena^®^ feed were available ad libitum.

After a week of acclimating to the light–dark schedule, 20 min of light was gradually added each day for 17 days to reach a 16:8 light–dark cycle for photostimulation and egg production. Chemical exposure to Fomtec Enviro USP began following the photostimulation period and continued for 60 days. Eggs were collected, incubated, and hatched in a 1502 Digital Sportsman Incubator and a 1550 Digital Hatcher (GQF Manufacturing). The chicks were housed together for 21 days in a Universal Box Brooder. The chicks were fed Purina Game Bird 30% Protein Starter^®^ and deionized water ad libitum. After the 60-day study and 21-day post-emergence for the chicks, all birds were euthanized through carbon dioxide asphyxiation followed by cervical dislocation as the form of euthanasia approved by the IACUC protocol. Treatment birds were kept at −25 °C until necropsy, tissue residue analyses, lipid analyses, and other purposes, as needed.

### 2.2. Test Chemicals, Exposure Verification, and Average Daily Intake (ADI)

Fomtec Enviro USP was obtained from the research sponsor and stored in the dark at room temperature until use. Initially, the Safety Data Sheet was used to identify the chemical components in the test foam. This strategy helped resolve the identity of individual chemicals or chemical classes by CAS number and the range of % compositions for the respective chemicals or classes (Supplementary Materials Table S1). We next purchased neat materials of the identified chemicals or neat chemicals within the identified chemical classes. Finally, we used those chemicals as standards to support targeted LC-MS analysis of exposure solutions and tissue residues.

Stocks of dosing solutions were prepared in distilled water. The stock solutions were used to refill bird water bottles as needed throughout the study. Concentrations of Fomtec Enviro USP were prepared at low (FL), medium (FM), and high concentrations (FH) (0.01%, 0.1%, and 0.25%, respectively). Foam products were a 3% solution (the typical application rate for AFFFs and MIL-SPEC expectations for new foams). Our Fomtec exposure concentrations were selected using the results of an earlier water consumption trial with a fluorinated foam. We observed that birds exposed to 0.5% Buckeye Platinum Plus C6 in water consumed 19% less than control birds. Based on the avian reproduction literature [15], we hypothesized that this reduced water consumption might have a detrimental effect on egg production, so we selected foam concentrations for our chronic exposure study below 0.5%.

As the study used the oral route of exposure, water was precisely measured to determine the amount of dosing solution ingested per quail pair. The total volume of water per bird was computed and used to determine the average daily intake (ADI), calculated by the amount of water consumed (a), the chemical concentration (c), the average weight of the bird (w), and the number of days exposed to treatment water (e), resulting in a single value of mg toxicant × kg^−1^ body weight × day^−1^ (Equation (1)).

(1) $$ADI=\frac{\frac{\left(a\times c\right)}{w}}{e}$$

### 2.3. Instrumental Analyses and Quality Control

LC-MS/MS was used to quantify the concentrations of the treatment stock solutions and in tissue residue analyses (described below). Liquid chromatography (UltiMate 3000 UHPLC+ Thermo Scientific, Waltham, MA, USA) coupled to a mass spectrometer ([MS/MS]: Thermo Scientific TSQ Endura Triple Stage Quadrupole) was used to quantify SDS and DGMBE in residue extracts and water samples. To quantify SDS, electrospray ionization (ESI) was used together with a positive ion source after optimizing parameters for ESI as well as MS for SDS and TCPP (internal standard from Sigma Aldrich, Saint Louis, MO, USA) with full scan and selected reaction monitoring (SRM) modes. Following optimization, 20 µL of the sample was auto-injected onto a Phenomenex Kinetex^®^ (2.6 µm C18, 50 × 2.1 mm) column within an oven maintained at 35 °C. Mobile phases included (A) 50 mM formic acid in water (LC-MS-grade) and (B) acetonitrile (LC-MS-grade) at 0.3 mL/min. The run time for each sample was 15 min.

For DGMBE determination, atmospheric pressure chemical ionization (APCI) with a positive ion source was used in full scan and SRM. First, 20 µL of the sample was injected into a Thermo Scientific Hypersil Gold (1.9 µm C18, 100 × 2.1 mm) column in a 40 °C column oven. Mobile phases included (A) 0.1% formic acid in water (LC-MS-grade) and (B) 0.1% formic acid in acetonitrile (LC-MS-grade) at 0.25 mL/min. The run time for each sample was 15 min.

Solvent blanks (LC-MS-grade methanol) and quality control (QC) standards (5 or 10 ng/mL for SDS, 100 or 250 ng/mL for DGMBE) were run after 5 to 10 samples as continuing calibration check standards. We found that more frequent blanks and QC standards prevented carryover and contamination and ensured that the instrument remained within calibration limits. Standard calibration curves included six data points plotted to produce a linear or quadric regression with an R^2^ of 0.995 or higher.

### 2.4. Tissue Extraction Methods

The extraction method for liver, egg, and feed samples (described below) was developed using a process similar to the Quick, Easy, Cheap, Effective, and Safe (QuEChERS) method for the extraction of biological media [16,17]. The protocols optimized for the liver, egg, and feed were also used to prepare experimental samples, the laboratory-fortified sample matrix (LFSM), and laboratory-fortified blanks (LFBs). Seven fortified replicate samples were prepared to determine the minimum reporting level (MRL), the detection limit (DL), and the percent recovery (% R). Liver, egg, and feed LFBs were made from subsets of control samples or feed samples for SDS. Liver and egg LFBs for DGMBE were prepared from chicken liver and chicken egg surrogates. LFBs were spiked to be within the bounds of the calibration curve; for SDS, LFBs were spiked to 100 ppb, and for DGMBE, 250 ppb.

### 2.4.1. Liver

All adult livers were extracted along with 15 chick livers from each treatment and control. Whole livers from chicks and adults were collected and placed into 15 mL or 50 mL polypropylene centrifuge tubes (Fisher Scientific, Waltham, MA, USA), respectively. For each liver sample, a subsample of ~0.25 g (ww) was excised and placed in 50 mL polypropylene centrifuge tubes (Fisher Scientific). Then, 20 µL of IS (TCPP, 10 µg/mL in acetonitrile) was added to each sample. Samples were vortexed to ensure homogenization before allowing the liver samples to sit for 30 min. After 30 min, LC-MS-grade water (Optima^®^) was added to the sample and vortexed for 30 s. QuEChERs salts (4 g of magnesium sulfate, 1 g of sodium chloride; Fisher Scientific) were added to each liver sample and shaken vigorously before vortexing for 30 s. Samples were centrifuged at 1000× *g* for 5 min at 0 °C. After centrifugation, 5 mL of the supernatant was transferred into a glass test tube (16 × 100 mm). After that, the samples were evaporated to dryness under nitrogen at 35° C. Samples were reconstituted with 1 mL of methanol (Optima^®^ LC-MS-grade) and vortexed. Homogenate was filtered with a 0.2 µ cellulose acetate filter (Thermo Scientific). Samples were transferred into 2 mL autosampler vials (Fisher Scientific). 

### 2.4.2. Eggs

We placed the egg without the shell into a 50 mL polypropylene centrifuge tube (Fisher Scientific). Eggs were shaken and vortexed to homogenize them; the extraction procedure for eggs was similar to liver extraction, except ~2 g (ww) of the egg sample was used.

### 2.4.3. Feed

The study used two preferred feeds: Purina^®^ Game Bird Layer for adults and Purina^®^ Game Bird 30% Protein Starter for chicks. Throughout the study, samples of multiple bags of feed were set aside for subsequent residue analyses. Extractions were prepared the same way as liver extractions, except ~2 g of feed was used; water (Optima^®^ LC-MS-grade) was used as the extraction solvent for feed.

### 2.5. Gravimetric Lipid Determination

Lipids were extracted and examined to determine lipid content in adult and chick livers after parental exposure to Fomtec Enviro USP. The lipid procedure followed a modified version of published methods [18,19,20]. Lipid extraction was performed on all livers from adults and 10 livers from chicks in each treatment group, including control birds. A sample (~1 g for chicks and ~1.5 g for adults) was obtained and then placed in 16 × 125 mm (Thermo Scientific) glass test tubes, and 2:1 methanol (Optima^®^ LC-MS-grade):methylene chloride (HPLC-grade), both Fisher Scientific, Waltham, MA, USA, was added. Livers were manually minced with a glass rod and then allowed to sit for 30 min before centrifugation (1000× *g*, 5 min, 0 °C). The extract was filtered (0.2 µ PTFE) and then dispensed into a pre-weighed metal pan and evaporated for 20 h before re-weighing.

### 2.6. Chronic Toxicity Values (CTVs)

CTVs were estimated based on dose-dependent NOEL and LOAEL for SDS and DGMBE for species-specific and tissue-specific values. In addition, any reproductive or residue data that differed significantly between treatment groups were identified and evaluated for the NOEL and LOAEL. As a result, the CTVs can be considered conservative in an effort to protect the most sensitive population groups.

### 2.7. Endpoints and Statistics

The endpoints of this chronic toxicity study included reproduction, growth, and survival over the 60-day exposure time for adults and 21 days for chicks. Additional endpoints were weight change, the number of eggs laid, the average arrest day in embryonic development (unhatched eggs), the pipped proportion, hatching success, chick weight (hatch, 7 d, 14 d, 21 d), growth rate, and survival. Data obtained were analyzed using JMP statistical software (Version 17.2) with α = 0.05. For parametric statistical testing, ANOVA was used to compare groups for the number of eggs laid, the average embryo arrested development date, chick hatching weight, and biometric characteristics. If there was a significant treatment effect, a Tukey–Kramer post hoc test was used. Nonparametric data were analyzed using the Kruskal–Wallis test, with Dunn’s test for post hoc analysis. 

## 3. Results and Discussion

### 3.1. Average Daily Intake (ADI)

The average daily intake for SDS and DGMBE (two out of four primary components in Fomtec Enviro USP) was calculated for each bird and averaged for each treatment concentration using Equation (1) (Table 1). The ADI for nominal Fomtec exposure solutions was 14 ± 0.4, 156 ± 4, and 394 ± 7 mg/kg/day. The ADI for SDS was 0.05 ± 0.001, 0.15 ± 0.01, and 0.32 ± 0.01 mg per kg BW per day for FL, FM, and FH, respectively. The ADI for DGMBE was 0.49 ± 0.01, 6.5 ± 0.28, and 18.4 ± 0.37 mg per kg BW per day (FL, FM, and FH, respectively) (Table 1). The survival rate for adults in the study was 97%, which is above the criteria (90%) for study validity [14,21].

Throughout the 60-day chronic toxicity study, water consumption monitoring and measurement provided valuable information on ingestion levels and Fomtec exposure per pair. During the breeding season, water consumption increases to provide the water needed for egg production and the overall increase in nutrients required for successful breeding. Therefore, water consumption can affect reproductive parameters during the breeding season, including egg laying, the viability of eggs, offspring survival, size, and overall growth.

### 3.2. Adult Weight Change

Adults used in this study were of breeding age (>8 weeks) but still young, and they continued to grow and gain weight throughout the study. As the breeding season began after photostimulation, females were at their heaviest weight to account for increased water consumption for egg production. Overall, there was an increase in average weight from initial weight to post-exposure weight in both females (39.9 ± 5.3 g) and males (34.4 ± 5.1 g).

In comparing the initial weight of all females and all males using ANOVA, there was no significant difference in weight among the treatment groups (*p* = 0.423, *p* = 0.132). Comparison of the final weights of females and males revealed a significant difference for females but not males (ANOVA; *p* = 0.043, *p* = 0.348). A significant difference was observed between the initial weight and the final weight for control females (*p* < 0.0001) and between the initial and final weight for the high Fomtec exposure females (*p* < 0.0001); a significant difference was not observed between initial and final weight in the low and medium exposure groups. The Fomtec, SDS, and DGMBE NOEL were 14.2, 0.05, and 0.48 mg/kg/d, respectively. We calculated the LOAEL based on the change in female weight from pre-exposure to post-exposure for Fomtec, SDS, and DGMBE, which was 394, 0.32, and 18.4 mg/kg/d, respectively.

Water consumption was compared to see if one treatment group consumed more of the Fomtec Enviro USP, which could have impacted the final weight of female birds. We found no significant difference in water consumption among the control and exposure groups (*F*_3,14_ = 2.943, *p* = 0.080) (Table 1). Another potential factor affecting the final weight of females could have been the average number of eggs laid per day. We observed a significant difference in the number of eggs laid per day among the control and exposure groups (*F*_3,14_ = 12.826, *p* = 0.0007) (Table 2). In a post hoc test, a significant difference in the average number of eggs laid per day occurred between the low exposure group and control birds, the medium exposure group, and the high exposure group (*p* = 0.0004, *p* = 0.0116, and *p* = 0.0028, respectively) (Table 2). The significant difference in final weights for control and high exposure group females was unrelated to the difference observed in the average number of eggs laid per day. Factors other than water consumption and eggs laid could impact weight gain, leading to the significant differences we observed. 

### 3.3. Egg Collection, Incubation, and Hatching Success

A total of 759 eggs were laid throughout the 60-day study, ranging from 28 to 59 eggs laid per pair, depending on the treatment group (Table 2). There were only two cracked eggs in the entire study; one was laid by a female control bird, and one came from an exposed female bird (FM). One-way ANOVA was used to compare the average number of eggs laid per day among the control and treatment groups. The average number of eggs laid per day was 0.85 ± 0.05, which is within the expected values for reproductive parameters [21]. There was a significant difference in the number of eggs laid per day per hen (*F*_3,14_ = 12.77, *p* = 0.0007). A post hoc test was conducted to compare differences between treatments. Results indicated a significant difference between the low exposure group and control birds, the medium exposure group, and the high exposure group (*p* = 0.0004, *p* = 0.0028, and *p* = 0.0116, respectively).

A subset (293 out of 759 eggs) was placed into the incubator at various time points throughout the study. Chicks hatched between days 22 and 24 in the hatcher and remained there for 12–39 h after hatching. Food and water were not provided to chicks in the hatcher, as chicks can maintain nutrients from the yolk sac absorbed during the final days of development [22]. A total of 235 chicks hatched during the study, and 206 of those chicks survived for 21 days after hatching (Table 2). The overall hatching success rate was 80 ± 3.6% among all of the treatment groups. A hatching success rate of 80% is within the range (75–90%) of normal reproductive parameters for birds [21]. There was no significant difference in hatching success between treatment and control groups (*F*_3,14_ = 1.471, *p* = 0.276). 

### 3.4. Mean Arrested Embryo Development Day and Percentage of Infertile Eggs

Following the 24th day of incubation, eggs that did not hatch were opened to examine the development stage of the embryo [23]. The average arrest day, including eggs laid by control and treatment birds, was 16.2 ± 1.1 (Table 2). Eggs that were infertile or without visible embryonic development were not part of the calculated arrest day average. There was not a significant difference between the average arrest day for treatment and control groups (*F*_3,34_ = 0.262, *p* = 0.852). Furthermore, the proportion of infertile eggs from exposed parents compared to eggs from control parents was compared using ANOVA; there was no significant difference in the percentage of infertile eggs among the exposed and control groups (*F*_3,14_ = 0.116, *p* = 0.949). 

### 3.5. Pipped Only Proportion

The “pipped only proportion” was calculated based on chicks that pipped (began hatching by forming an initial chip in the egg) but were ultimately unsuccessful at hatching. We used one-way ANOVA to compare the proportion of chicks that pipped among the treatment groups; the treatment effect was not significant (*F*_3,14_ = 1.76, *p* = 0.213),

### 3.6. Juvenile Survival

All chicks hatched were housed in the same brooder under similar conditions from day 1 to day 21 of growth until euthanasia. The juvenile survival rate was 88 ± 3% for chicks from control and treatment parents (Table 2). There was no significant treatment effect on chick survival (*F*_3,14_ = 0.234, *p* = 0.871).

### 3.7. Chick Growth

Chicks were weighed immediately after hatching and then again on days 7, 14, and 21; euthanasia occurred on day 21. ANOVA was used to evaluate if parental exposure to Fomtec Enviro USP caused a difference in chick weight; no significant differences were observed in chick weights at hatch or at 7, 14, or 21 days (*p* = 0.179, *p* = 0.449, *p* = 0.429, and *p* = 0.484, respectively). 

### 3.8. Adult Biometrics, Relative Liver Weights, and Liver Lipid Content

Biometric data for adult birds included the length of the wings, head, and tarsals for females and males (Supplementary Materials Table S2). There was no significant difference in left or right wing size for females or males. Furthermore, there was no significant difference in head size for females or males (*p* = 0.497, *p* = 0.639) among exposed birds compared to control birds. Regarding the length of tarsals, there was a significant difference for the left tarsal in female birds (*F*_3,14_ = 5.747, *p* = 0.013). According to a post hoc test, high exposure birds had larger left tarsals than control birds (*p* = 0.0078). For left tarsals in males and right tarsals in females and males, there was no significant difference in size (*p* = 0.445, *p* = 0.471, *p* = 0.461, respectively). Tarsals are essential for holding on to a roost, collecting food, and protection from predators. In addition, as much as 25% of a quail diet can be composed of insects and other invertebrates; birds use their tarsals to scratch at leaf litter on the ground to expose insects and other food sources.

Liver weight was not significantly different between control and Fomtec-exposed birds (*F*_3,29_ = 0.455, *p* = 0.716). Relative liver weight (liver weight/bird weight) was not different between control and treatment birds (*F*_3,29_ = 0.078, *p* = 0.971). In addition, there was no significant difference in liver lipid content for females or males (*p* = 0.113, *p* = 0.224, respectively).

### 3.9. Chick Biometrics, Relative Liver Weights, and Liver Lipid Content

Biometric parameters were measured to compare the length of the head, wings, and tarsals of chicks at 21 d (Supplementary Materials Table S3). In addition, relative liver weight and lipid content were also evaluated for differences between treatment groups. There was not a significant difference in chick head size (*F*_3,196_ = 0.962, *p* = 0.4117) or liver lipid content (*F*_3,79_ = 1.213, *p* = 0.311) between control and treatment birds. There were significant differences in left wing (*F*_3,196_ = 8.91, *p* < 0.0001), right wing (*F*_3,196_ = 12.30, *p* < 0.0001), left tarsal (*F*_3,196_ = 15.063, *p* = < 0.0001), right tarsal (*F*_3,196_ = 19.608, *p* < 0.0001), and liver weight (*F*_3,196_ = 7.918, *p* < 0.0001) between chicks from control parents and chicks from exposed parents.

Chicks from FH parents had larger livers in comparison to chicks from FL parents (*p* < 0.0001) and chicks from control parents (*p* = 0.0176) but smaller livers than chicks from FM parents (*p* = 0.0048). Relative liver weight differed significantly between chicks from control and treatment parents (*F*_3,195_ = 8.529, *p* < 0.0001). Chicks from FH and FM parents had higher relative liver weights compared to chicks from control and FL parents. Interestingly, liver lipid content was not affected by the changes in liver mass. 

The NOEL for the left wing in chicks was calculated from ADI dosing solution concentrations of nominal Fomtec, SDS, and DGMBE (14.3, 0.05, and 0.48 mg/kg/d, respectively) (Table 3). The LOAEL for the left wing was calculated based on the ADI for Fomtec, SDS, and DGMBE concentrations (155.6, 0.15, and 6.5 mg/kg/d, respectively) (Table 3). For the right wing, the left and right tarsal, and chick liver weight, the LOAEL was the same and calculated based on the ADI of nominal Fomtec, SDS, and DGMBE dosing concentration (14.3, 0.05, and 0.48 mg/kg/d, respectively) (Table 3).

### 3.10. Residue Analysis

#### 3.10.1. Adult and Chick Liver

Adult and chick livers were extracted for residue analysis of two chemicals of interest in Fomtec Enviro USP: SDS and DGMBE. There was a significant difference in the amount of SDS (*F*_3,28_ = 6.458, *p* = 0.002) and DGMBE (*F*_3,29_ = 7.622, *p* = 0.0008) in adult livers. After a post hoc analysis, the FH group had an SDS concentration of 2015 ng/mL, which was significantly higher in the liver than the control (*p* = 0.0029), FL (*p* = 0.0028), and FM (*p* = 0.0437) exposure groups (Table 4). Chick liver SDS concentrations were not significantly different among treatment and control groups (*F*_3,58_ = 0.693, *p* = 0.560) (Table 4). The source of SDS is likely the dosing solution, which ranged in concentration from 341.7 to 2014.5 ng/ mL. The concentration of SDS in feed for adults and chicks was 2.80 ng/g, which would not have increased exposure to such a level as to cause significant differences between treatment groups.

DGMBE concentrations in adult livers differed significantly between treatment groups (*F*_3,29_ = 7.62, *p* = 0.0008). Control livers had more DGMBE compared to FL (*p* = 0.0044) and FM (*p* = 0.0061); there was also a significant difference between FH and FL (*p* = 0.0403). As for chicks, there was a difference between treatment groups (*F*_3,58_ = 12.29, *p* < 0.0001), and control chicks had significantly more DGMBE compared to FL (*p* = 0.0011), FM (*p* = 0.0004), and FH (*p* < 0.0001) (Table 4). The birds could have been exposed through the dosing solutions or through the feed; adult feed (Purina^®^ Game Bird Breeder Layena^®^) had a DGMBE concentration of 102 ng/mL, and chick feed (Purina Game Bird 30% Protein Starter^®^) had a DGMBE concentration of 80 ng/mL. 

#### 3.10.2. Whole Egg

SDS and DGMBE were found in egg samples; there was a significant treatment effect for SDS (*F*_3,59_ = 38.72, *p* < 0.0001) and DGMBE (*F*_3,58_ = 25.70, *p* < 0.0001). The SDS concentration in eggs of the FH exposure group was significantly higher than that of control eggs (*p* < 0.0001), FL (*p* < 0.0001), and FM (*p* < 0.0001) (Table 4). SDS is maternally transferred from adults to the egg after exposure via drinking water. DGMBE also had the highest concentration in eggs from the FH exposure group, with significant differences compared to control (*p* < 0.0001), FL (*p* < 0.0001), and FM (*p* = 0.0012) eggs (Table 4). Also, eggs from the FM exposure group had significantly higher levels of DGMBE compared to control (*p* = 0.0043) and FL eggs (*p* = 0.0080) (Table 4). 

## 4. Conclusions

Chronic avian toxicity testing was used to evaluate the potential impact of exposure to Fomtec Enviro USP, a fluorine-free AFFF. Reproductive parameters and residue analyses revealed significant differences between exposed birds and their offspring compared to control (untreated) birds. NOEL and LOAEL values were determined based on nominal Fomtec ADI as well as the two primary measured components in Fomtec (SDS and DGMBE). Fomtec-exposed birds laid fewer eggs/day than control birds and had greater changes in weight during the 60-day study. We were also able to calculate NOEL and LOAEL values for certain adult and chick biometrics; however, the potential implications of these effects on bird populations are less obvious than endpoints related to reproduction. Although the apparent natural occurrence of SDS and DGMBE complicates interpretation of the results, we observed clear increases in SDS and DGMBE concentrations in Fomtec-exposed birds and their offspring. This study was designed to collect baseline toxicology data (survival, growth, and reproduction) in an avian model regarding an emerging group of products for which there are very little data. Now that some baseline data have been collected, follow-up studies with a different experimental design could focus on the mechanisms involved in the observations we made. In addition, the possibility of impacts on male birds (for example, sperm quality) and the downstream implications for reproduction should also be investigated to complement this study, which heavily focused on female birds.

## Figures and Tables

**Table 1:** Exposure concentrations of SDS and DGMBE, mean body weight, mean water consumption, and average daily intake (ADI).

	Mean Body Weight ± SE (kg)	Mean Water Consumption ± SE (mL/Bird/Day)	ADI (mg/kg Body Weight/Day) ± SE
Control	0.245 ± 0.008	34.1 ± 1.2	NA
342 ng/mL SDS ^1^	0.223 ± 0.017	30.0 ± 2.3	0.05 ± 0.00
945 ng/mL SDS ^2^	0.224 ± 0.013	33.4 ± 1.6	0.15 ± 0.01
2015 ng/mL SDS ^3^	0.257 ± 0.009	37.4 ± 0.2	0.32 ± 0.01
3518 ng/mL DGMBE ^1^	0.223 ± 0.017	30.0 ± 2.3	0.49 ± 0.01
42,345 ng/mL DGMBE ^2^	0.224 ± 0.013	33.4 ± 1.6	6.5 ± 0.28
117,634 ng/mL DGMBE ^3^	0.257 ± 0.009	37.4 ± 0.2	18.4 ± 0.37

SDS = sodium dodecyl sulfate. DGMBE = diethylene glycol monobutyl ether. ^1^ 0.01% Fomtec exposure. ^2^ 0.1% Fomtec exposure. ^3^ 0.25% Fomtec exposure.

**Table 2:** Reproductive performance summary of NOBO (*Colinus virginianus*) from chronic (60-day) Fomtec Enviro USP exposure.

Reproductive Parameter	Control (n = 6)	FL (n = 3)	FM (n = 3)	FH (n = 3)
Eggs laid	351	85	154	169
Eggs set	102	52	68	71
Live 21 d embryos	82	43	56	54
Hatchlings/eggs set (%)	80	83	82	76
21-day-old survivors/hatchlings (%)	88	84	91	87
Mean hatch success (%)	80 ± 4.5	79 ± 8.6	83 ± 4.6	78 ± 16
Mean arrested development day	15.7 ± 1.9	15.1 ± 1.8	16.0 ± 3.4	18.0 ± 2.3
Average percentage infertile (%)	5.56	3.90	4.42	8.97
Juvenile survival rate (%)	89 ± 4.2	83 ± 12	91 ± 3.8	87 ± 9.5

**Table 3:** Chronic toxicity values for reproductive parameters in NBO. Adults were exposed over 60 days to three concentrations of Fomtec Enviro USP in drinking water. Chicks hatched from eggs laid by exposed adults, and they were monitored for growth over 21 days.

CTV Parameter	Fomtec ADI (mg/kg/day)		SDS ADI (mg/kg/day)		DGMBE ADI (mg/kg/day)	
	NOEL	LOAEL	NOEL	LOAEL	NOEL	LOAEL
Eggs laid/hen/day	NA	14.3	NA	0.05	NA	0.5
Wt change—female initial to final	14.2	394.2	0.05	0.3	0.5	18.4
Eggs laid/hen	NA	14.3	NA	0.05	NA	0.5
Adult left tarsal	14.2	394.2	0.05	0.3	0.5	18.4
Chick left wing	14.2	155.6	0.05	0.1	0.5	6.5
Chick right wing	NA	14.2	NA	0.05	NA	0.5
Chick left tarsal	NA	14.2	NA	0.05	NA	0.5
Chick right tarsal	NA	14.2	NA	0.05	NA	0.5
Chick liver	NA	14.2	NA	0.05	NA	0.5

**Table 4:** Tissue residues (ng/g ± standard error, SE) of sodium dodecyl sulfate (SDS) and diethylene glycol monobutyl ether (DGMBE) in adults exposed to Fomtec Enviro USP over 60 d and chicks (21 d) that hatched from eggs laid by exposed adults.

	Control		FL (0.01%)		FM (0.1%)		FH (0.25%)	
	SDS	DGMBE	SDS	DGMBE	SDS	DGMBE	SDS	DGMBE
Adult liver tissue	104 ± 53	1372 ± 94	55 ± 15	836 ± 104	170 ± 62	855 ± 117	450 ± 98	1302 ± 53
Offspring liver tissue	138 ± 27	1587 ± 97	98 ± 10	1045 ± 108	81 ± 7.4	103 ± 343	117 ± 60	794 ± 85
Whole egg	16 ± 1.1	570 ± 16	16 ± 1.5	587 ± 11	83 ± 21	851 ± 75	316 ± 41	1163 ± 84

SDS = sodium dodecyl sulfate. DGMBE = diethylene glycol monobutyl ether.

## Data Availability

Data for this project are available from the corresponding author upon request.

## Supplementary Materials

The following supporting information can be downloaded at: https://www.mdpi.com/article/10.3390/toxics13060474/s1, Table S1: Chemical components in the foam identified by Safety Data Sheet or chemical analysis; Table S2: Exposure concentrations of SDS and DGMBE, mean body weight, mean water consumption, and average daily intake (ADI); Table S3: Reproductive performance summary of NOBO (*Colinus virginianus*) from chronic (60-Day) Fomtec Enviro USP exposure; Table S4: Adult NOBO biometric data including wings, head, tarsals, liver weight, and relative liver weight (± SE); Table S5: NOBO chick biometric data including wings, head, tarsals, and liver weight (± SE).
