# Supplementary material for: Chronic Reproductive Toxicity of Fomtec Enviro USP, a Fluorine-Free Firefighting Foam, to Northern Bobwhite (Colinus virginianus)
Source: Toxics. 2025 Jun 3;13(6):474. doi: 10.3390/toxics13060474 (PMC12197796; doi:10.3390/toxics13060474)
Supplement: Supplementary file 1 [file toxics-13-00474-s001.zip › toxics-3629285-supplementary.pdf]

Supplemental Information

Table S1. Chemical components in the foam identified by Safety Data Sheet or chemical analysis.

Foam	
**Fomtec Enviro USP**	
Diethylene glycol monobutylether (112-34-5)	5-10%
C12-C14 sulfuric acid esters (9058-18-9)	5-10%
C12-C14 alcohols ethoxylated sulfates (68891-38-3)	1-3%
C12-C14 amines N-oxides (308062-28-4)	<1%

Table S2. Exposure concentrations of SDS and DGMBE, mean body weight, mean water consumption, and average daily intake (ADI).

Verified Exposure Concentration	Sex	Mean body weight ± SE (kg)	Mean water consumption ± SE (mL/ bird/ day)	ADI (mg/kg body weight/day) ± SE
**Control**	M	0.233 ± 0.009	34.1 ± 1.2	NA
	F	0.256 ± 0.007		
**342 ng/mL SDS[1]**	M	0.232 ± 0.020	30.0 ± 2.3	0.045 ± 0.00
	F	0.213 ± 0.013		0.048 ± 0.00
**945 ng/mL SDS[2]**	M	0.218 ± 0.006	33.4 ± 1.6	0.15 ± 0.01
	F	0.230 ± 0.020		0.14 ± 0.01
**2015 ng/mL SDS[3]**	M	0.254 ± 0.014	37.4 ± 0.2	0.31 ± 0.01
	F	0.260 ± 0.003		0.32 ± 0.00
**3518 ng/mL DGMBE[1]**	M	0.232 ± 0.020	30.0 ± 2.3	0.47 ± 0.01
	F	0.213 ± 0.013		0.50 ± 0.02
**42345 ng/mL DGMBE[2]**	M	0.218 ± 0.006	33.4 ± 1.6	6.65 ± 0.28
	F	0.230 ± 0.020		6.43 ± 0.27
**117634 ng/mL DGMBE[3]**	M	0.254 ± 0.014	37.4 ± 0.2	18.28 ± 0.71
	F	0.260 ± 0.003		18.46 ± 0.03

SDS = Sodium dodecyl sulfate

DGMBE = Diethylene glycol monobutyl ether

[1]0.01% Fomtec exposure

[2]0.1% Fomtec exposure

[3]0.25% Fomtec exposure

Table S3. Reproductive performance summary of NOBO (*Colinus virginianus*) from chronic (60-Day) Fomtec Enviro USP exposure.

Reproductive Parameter	Control (n=6)	FL (n=3)	FM (n=3)	FH (n=3)
**Eggs Laid**	351	85	154	169
**Eggs Set**	102	52	68	71
**Viable Embryos**	97	50	65	64
**Live 21-d embryos**	82	43	56	54
**21-day old Survivors**	72	36	51	47
**21-day old Survivors/hen ± SE**	12 ± 1	12 ± 5	17 ± 1	16 ± 4
**Eggs laid/hen ± SE**	59 ± 0.8	28 ± 9.0	51 ± 4.3	56 ± 1.8
**Eggs laid/hen/day ± SE**	0.98 ± 0.01	0.51 ± 0.13	0.86 ± 0.07	0.94 ± 0.03
**Viable embryos/eggs set**	95%	96%	96%	90%
**Live 21-d embryos/viable embryos**	85%	86%	86%	84%
**Hatchlings/eggs set**	80%	83%	82%	76%
**21- day old survivors/hatchlings**	88%	84%	91%	87%
**21-day old survivors/eggs set**	71%	69%	75%	66%
**Mean Hatch Success (%)**	80 ± 4.5	79 ± 8.6	83 ± 4.6	78 ± 16
**Mean arrested development day**	15.7 ± 1.9	15.1 ± 1.8	16.0 ± 3.4	18.0 ± 2.3
**Pipped-only probability**	4.52 ± 2.90	0.00 ± 0.00	8.46 ± 6.32	1.28 ± 1.28
**Average Percentage Infertile (%)**	5.56	3.90	4.42	8.97
**Pipped but not hatched percentage (%)**	4.52 ± 2.9	0.00 ± 0.00	8.46 ± 6.3	1.28 ± 1.28
**Juvenile Survival Rate (%)**	89 ± 4.2	83 ± 12	91 ± 3.8	87 ± 9.5
**Adult wt change (g)**	22.6 ± 6.2	8.0 ± 7.4	14.9 ± 4.8	33.9 ± 7.2
**Female wt change (g)**	26.4 ± 10.8	1.9 ± 1.1	18.2 ± 10.5	42.3 ± 24.4
**Male wt change (g)**	18.9 ± 7.7	14.2 ± 8.2	11.6 ± 6.7	25.5 ± 14.7

Table S4. Adult NOBO biometric data including wings, head, tarsals, liver weight, and relative liver weight (± SE).

Treatment	Sex	Left wing (mm)	Right Wing (mm)	Head (mm)	Left Tarsal (mm)	Right Tarsal (mm)	Liver Weight (g)	Relative Liver Weight (mg)
**Control**	Female (n=6)	107.40 ± 3.19	103.44 ± 3.21	39.42 ± 0.57	37.65 ± 0.80	36.30 ± 0.71	10.34 ± 0.82	40.40 ± 3.27
	Male (n=6)	106.93 ± 3.18	97.01 ± 4.64	42.01 ± 0.39	37.62 ± 0.99	38.44 ± 1.52	4.93 ± 0.21	21.32 ± 1.05
**FL (0.01%)**	Female (n=3)	91.04 ± 8.10	83.89 ± 5.24	39.83 ± 0.69	38.92 ± 0.90	37.27 ± 0.21	6.72 ± 0.96	31.30 ± 3.01
	Male (n=3)	104.41 ± 4.41	104.84 ± 5.24	40.44 ± 2.99	39.22 ± 1.25	36.31 ± 1.60	6.48 ± 1.89	27.19 ± 5.84
**FM (0.1%)**	Female (n=3)	101.80 ± 2.11	96.90 ± 6.24	38.90 ± 0.69	38.04 ± 0.89	37.33 ± 0.70	8.77 ± 1.18	38.01 ± 3.63
	Male (n=3)	99.94 ± 3.00	102.55 ± 0.95	41.04 ± 0.54	39.63 ± 0.97	38.67 ± 0.33	4.69 ± 0.25	21.52 ± 1.51
**FH (0.25%)**	Female (n=3)	103.63 ± 3.34	102.15 ± 6.86	40.36 ± 0.11	40.13 ± 1.63 *	40.27 ± 0.56	10.64 ± 0.91	41.02 ± 3.98
	Male (n=3)	95.26 ± 5.51	104.81 ± 1.26	42.90 ± 1.24	40.93 ± 0.34	40.15 ± 0.37	5.81 ± 0.80	22.73 ± 2.20

* = Significantly different from control birds

Table S5. NOBO chick biometric data including wings, head, tarsals, and liver weight (± SE).

	**Control (n = 63)**	**FL (n = 35)**	**FM (n = 50)**	**FH (n = 45)**
**Left Wing (mm)**	71.48 ± 0.57^{A}	69.80 ± 0.76^{A}	66.97 ± 0.85^{B}	66.60 ± 1.19^{B}
**Right Wing (mm)**	72.82 ± 0.48^{A}	70.01 ± 0.64^{B}	68.50 ± 0.77^{B}	68.92 ± 0.62^{B}
**Head (mm)**	29.44 ± 0.19	29.49 ± 0.39	28.87 ± 0.40	28.99 ± 0.33
**Left Tarsal (mm)**	29.14 ± 0.78^{A}	25.32 ± 0.30^{B}	24.97 ± 0.29^{B}	25.47 ± 0.23^{B}
**Right Tarsal (mm)**	28.85 ± 0.79^{A}	25.13 ± 0.34^{B}	24.10 ± 0.22^{B}	24.46 ± 0.26^{B}
**Liver Weight (g)**	2.212 ± 0.040^{A}	2.039 ± 0.049^{A}	2.318 ± 0.07^{B}	2.427 ± 0.062^{B}
**Relative Liver Weight**	0.043 ± 0.0007^{A}	0.041 ± 0.0012^{A}	0.046 ± 0.0009^{B}	0.047 ± 0.0008^{B}
